# Cost, affordability and cost-effectiveness of strategies to control tuberculosis in countries with high HIV prevalence

**DOI:** 10.1186/1471-2458-5-130

**Published:** 2005-12-12

**Authors:** Christine SM Currie, Katherine Floyd, Brian G Williams, Christopher Dye

**Affiliations:** 1 School of Mathematics, University of Southampton, Southampton, SO17 1BJ, UK; 2 HIV/AIDS, Tuberculosis and Malaria cluster, World Health Organization, 20 Avenue Appia, CH-1211 Geneva 27, Switzerland

## Abstract

**Background:**

The HIV epidemic has caused a dramatic increase in tuberculosis (TB) in East and southern Africa. Several strategies have the potential to reduce the burden of TB in high HIV prevalence settings, and cost and cost-effectiveness analyses can help to prioritize them when budget constraints exist. However, published cost and cost-effectiveness studies are limited.

**Methods:**

Our objective was to compare the cost, affordability and cost-effectiveness of seven strategies for reducing the burden of TB in countries with high HIV prevalence. A compartmental difference equation model of TB and HIV and recent cost data were used to assess the costs (year 2003 US$ prices) and effects (TB cases averted, deaths averted, DALYs gained) of these strategies in Kenya during the period 2004–2023.

**Results:**

The three lowest cost and most cost-effective strategies were improving TB cure rates, improving TB case detection rates, and improving both together. The incremental cost of combined improvements to case detection and cure was below US$15 million per year (7.5% of year 2000 government health expenditure); the mean cost per DALY gained of these three strategies ranged from US$18 to US$34. Antiretroviral therapy (ART) had the highest incremental costs, which by 2007 could be as large as total government health expenditures in year 2000. ART could also gain more DALYs than the other strategies, at a cost per DALY gained of around US$260 to US$530. Both the costs and effects of treatment for latent tuberculosis infection (TLTI) for HIV+ individuals were low; the cost per DALY gained ranged from about US$85 to US$370. Averting one HIV infection for less than US$250 would be as cost-effective as improving TB case detection and cure rates to WHO target levels.

**Conclusion:**

To reduce the burden of TB in high HIV prevalence settings, the immediate goal should be to increase TB case detection rates and, to the extent possible, improve TB cure rates, preferably in combination. Realising the full potential of ART will require substantial new funding and strengthening of health system capacity so that increased funding can be used effectively.

## Background

**Figure 1:**
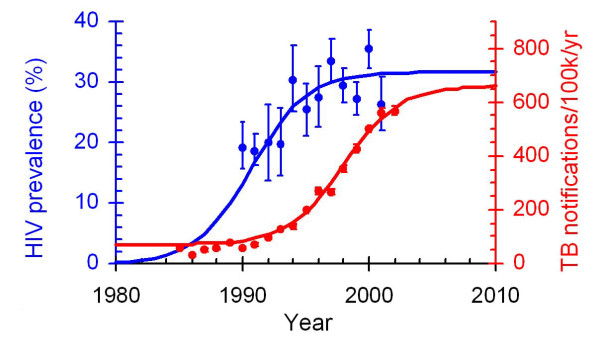
Trends in HIV prevalence and TB notifications in Kisumu District, Kenya Source: personal communication, John Mansoer (National TB programme, Kenya) and Laurence Mareum (Centers for Disease Control, Kenya).

There are an estimated 8.8 million new cases of tuberculosis (TB) each year, 1.7 million of which result in death[1]. In East and southern Africa in particular, the spread of HIV infection has led to a dramatic increase in TB notifications (Figure 1), and at national level up to 60% of TB patients are co-infected with HIV[2]. The prevalence of HIV is now one of the best predictors of national TB notification rates[1]. Various targets have been established for the control of TB and HIV/AIDS. The World Health Organization (WHO) global targets for TB control are to detect 70% of smear positive cases and to successfully treat 85% of those that are detected by 2005[3]. The Millennium Development Goals include targets to halve the year 1990 number of prevalent TB cases and TB deaths by 2015[4]. More recently the WHO has set a target to enrol 3 million people on antiretroviral therapy (ART) by the end of 2005 (the "3-by-5" Initiative); this would ensure that ART is provided to half of the estimated six million people who would otherwise be expected to die from HIV/AIDS in 2004 and 2005[5].

Several strategies have the potential to reduce the burden of TB in high HIV prevalence settings[6], but where budgets are constrained decisions must be made as to how they should be prioritized. Reviews of the cost and cost-effectiveness of interventions to control HIV/AIDS[7] and TB [8,9]show that previous studies are limited in three ways. First, most studies consider one intervention in one setting; different interventions are not compared in the same setting. Second, the impact of interventions on the transmission of HIV and TB is considered in only a few studies. Where transmission has been considered, different models have been used so that it is difficult to make fair comparisons among interventions. Third, the total number of people that need treatment and care if existing control targets are to be met, and the affordability of providing such treatment and care, has received little attention.

In a previous study[10], we used a dynamic model to compare the impact of a range of strategies on TB incidence and mortality in African countries with a high HIV prevalence. This model allows for the effect of strategies on TB transmission to be captured. Here, we use the model to compare the costs, effects, affordability and cost-effectiveness of a similar range of strategies, using data for Kenya. As far as possible, strategies are defined in relation to existing global targets or policy.

## Methods

Kenya is a low income country with a per capita income of US$390 in 2003, and a population of just under 32 million[1]. With almost 200,000 new cases of TB in 2003, Kenya ranks tenth globally in terms of total TB burden, and HIV prevalence among adult TB patients aged 15–49 is estimated at 29%[1]. Kenya has been implementing the internationally recommended TB control strategy known as DOTS[3] for more than ten years. We chose to focus our analysis on Kenya because the HIV and TB epidemics are similar to those in other countries in East Africa, data on HIV prevalence among women attending antenatal clinics are available for several districts over the past decade, and there are good TB notification data for all districts since 1985. There are also recent data on the cost of TB treatment[11], including the costs of initiatives to improve case detection and cure rates [1] (T. Pennas, written communication).

We considered seven strategies for reducing the burden of TB in Kenya and similar high HIV prevalence settings (Table 1). All strategies were assessed over the period 2004–2023, and compared with a "baseline" scenario in which interventions continue at their 2003 levels, which we take to be nationwide implementation of the DOTS strategy, a 50% TB case detection rate, a 70% TB cure rate, and no provision of antiretroviral treatment (ART) or treatment of latent TB infection (TLTI). While some people in Kenya were receiving ART or TLTI in 2003, no precise data are available and the numbers were small[5].

**Table 1:** The seven strategies for reducing the burden of TB and HIV that were evaluated

1) Improving TB case detection rates so that the WHO target of detecting 70% of new smear positive cases is reached in 2005 and then sustained 2) Improving TB cure rates so that the WHO target of 85% is reached in 2005 and then sustained 3) Improving TB case detection and cure rates simultaneously so that both WHO targets are met in 2005 and then sustained 4) Offering a six-month course of treatment for latent TB infection (TLTI) to people who are found to be HIV-positive but who do not have TB disease 5) TLTI as in strategy 4 but for life 6) Providing antiretroviral treatment (ART) to meet the target set by the WHO's "3-by-5" Initiative (with ART considered both for all in need and TB patients specifically) 7) Reducing HIV incidence through prevention programmes

**Figure 2:**
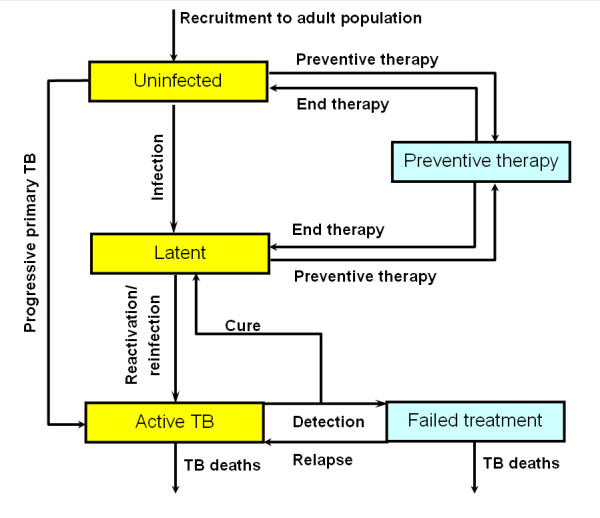
Outline of the tuberculosis (TB) sub-model. In the full model (see Methods, and supplementary material of [10]), active TB may be infectious or non-infectious, with movement allowed from active non-infectious disease to active infectious disease. An identical sub-model, with different parameter values, describes those with HIV. Death can occur in any state, but death rates are higher for patients with active disease.

We used a dynamic model of TB progression with a statistical model of HIV prevalence to assess the effectiveness of the different strategies. Figure 2 illustrates the general structure of the model, which was written in Visual Basic. A full description is available in the supplementary material for our previous study[10], including the parameter values and data sources used.

The TB model was designed so that the impact of strategies on TB transmission can be captured, and consists of two sub-models. The first describes the transitions between states for individuals who are not infected with HIV, or who are in the early stages of HIV (stages 1 and 2 of the WHO staging system[12,13]. The second describes transitions for those in the later stages of HIV. Active TB can arise when a) those who acquire a new TB infection develop progressive primary disease within 1 year or b) those who acquire a new TB infection enter a latent state, and may later develop TB by reactivation or reinfection. A proportion of individuals who are latently infected can develop TB within 1 year of reinfection. Active TB may be infectious or non-infectious.

A separate HIV model was used to estimate the number of new (incident) cases of HIV that occur in each time step (one time step is equal to one quarter of a year). After approximately 4 years (i.e. the time lag between HIV infection and late-stage HIV, defined here as WHO stage 3[12,14]), this number of individuals moves from the first TB sub-model (i.e. the sub-model that describes individuals without HIV infection and individuals in the early stages of HIV infection) to the corresponding state (either uninfected or latently infected with TB) in the second TB sub-model (i.e. the sub-model that describes individuals in the later stages of HIV infection). In the later stages of HIV infection, co-infection with TB leads to a greatly increased risk of developing TB, although a smaller fraction of those with active TB become infectious. Individuals with late-stage HIV infection (WHO stages 3 and above) also have a higher death rate, with and without active TB.

Estimates of HIV incidence were derived from estimates of the time dependent HIV prevalence and the survival function for HIV-infected people[15]. Estimates of HIV prevalence were obtained by fitting the available data on HIV prevalence over time to a double logistic function (see supplementary material to [10]). This allows the initial rate of increase, the peak prevalence, the final steady-state prevalence and the rate of convergence to the steady-state to be varied. Data from the US Bureau of Census show that in 1999 the prevalence of HIV infection among women attending antenatal clinics (ANC) in Kenya had reached about 14%[16]. Analysis of the most recent ANC data suggests that HIV prevalence had fallen to just over 9% by 2003[17]. A recent demographic and health survey indicates that HIV prevalence could have been as low as 7% in 2002[18]. Our main set of analyses assumed that the HIV prevalence was 14% in adults in 1999 and that it stabilises at this level. As part of uncertainty analyses, we also considered the consequences of assuming that HIV prevalence stabilizes at 7%.

The earlier version of our model[10] was designed to produce estimates of the number of TB cases and deaths that occur when different control strategies are implemented (and thus the number of TB cases and deaths that are averted by strategies that improve on the baseline scenario). For this study, we extended the model in three ways. First, we included as model outputs the number of TB patients detected and treated each year, the number of person years of ART and TLTI, the number of person years of treatment for AIDS-related opportunistic infections and the number of person years of palliative care for people with AIDS. We assumed that without ART people spend an average of 5.5 years in late stage HIV, and that during the last two years in this stage they have AIDS. We assumed that all HIV+ TB patients are eligible for ART, in line with WHO guidelines[14], and that overall 50% of those with AIDS receive ART, in line with the aim of the "3 by 5" Initiative.

Second, we incorporated data on the unit costs (per patient or per person year, as appropriate) in year 2003 US$ of detecting and treating TB, providing ART, providing a six month course of TLTI, providing treatment for AIDS-related opportunistic infections, and providing palliative care for people with AIDS. The unit costs, assumptions and sources of data are shown in Table 2[10,11,19-22]. We combined the number of patients or person years with unit costs to estimate total annual costs, and compared this with estimated annual government health expenditure in Kenya, which in 2000 was approximately US$200 million[23]. Costs incurred in future years were discounted at 3% per annum[24,25]. We only included costs to the health system because few data are available on the costs that are borne by patients and their households, particularly during TLTI and ART.

**Table 2:** Unit costs. For normal distributions, the first figure gives the mean and the second the variance. For uniform distributions, the two figures give the lower and upper limits.

Cost item	Unit	Unit Cost (2003 US$)	Uncertainty Distribution	Reference/assumptions
TB diagnosis costs, existing level of case detection	Sputum smear positive case detected	101	Normal (101, 25)	Nganda et al [11]. For every sputum smear positive case detected, assume 10 TB suspects are seen. For each TB suspect, assume 3 sputum smears and 1 chest X-ray are done, in line with WHO guidelines.
TB diagnosis when case detection rate increased to 70%	Sputum smear positive case detected	303	Uniform (202, 404)	Recent financial data from Kenya's applications to the Global Fund to fight AIDS, TB and Malaria (Pennas T, written communication), data submitted to WHO by Kenya and other high burden countries [1], and financial analyses for the forthcoming second Global Plan to Stop TB (2006–2015) being prepared by the Stop TB Partnership. These indicate that the average cost per sm+ patient detected will increase 2–4 times when activities to improve case detection rates to 70% are implemented. Further details from authors upon request.
Treatment for sputum smear positive TB cases, existing cure rate	Patient treated	140	Normal (140, 49)	Nganda et al [11].
Treatment for sputum smear negative TB cases, existing cure rate	Person treated	130	Normal (130, 43)	Nganda et al [11].
Treatment for sputum smear positive TB cases if cure rates improved to 85%	Person treated	280	Uniform (210, 350)	Recent financial data submitted to WHO by Kenya and other high burden countries [1], and financial analyses for the forthcoming second Global Plan to Stop TB (2006–2015) being prepared by the Stop TB Partnership. These indicate that the average cost per patient treated will double when activities to improve cure rates are implemented. Further details from authors upon request.
Treatment for sputum smear negative TB cases if treatment completion rates improved to 85%	Person treated	260	Uniform (195, 325)	As above for treatment of sputum smear positive cases.
TLTI (6 months)	Person treated	32	Uniform (27, 37)	Bell et al [19], WHO estimates of population coverage of HIV/AIDS interventions [20]. Assume 13% adult population accesses VCT each year [20], and that 36% are HIV+, 100% are screened for TLTI, 43% start treatment of whom 38% complete treatment [21].
TLTI (lifetime)	Person year of treatment	64	Uniform (54, 74)	As above for TLTI for six months, plus assumption that treatment for one year is double the cost of treatment for six months.
Treatment for AIDS-related opportunistic infections and palliative care in absence of ART	Person year of treatment	211	Uniform (167, 323)	Unit costs used for Kenya and other low-income high HIV prevalence countries in Africa in recent estimates of the resources needed for a comprehensive response to HIV/AIDS, prepared by UNAIDS (Gutierrez JP, written communication). Kenya is in the middle of the range.
Cost of ART for a TB patient	Six person months of treatment when TB and ART overlap	495	Uniform (420, 544)	Unit costs used for Kenya and other low-income high HIV prevalence countries in Africa in recent estimates of the resources needed for a comprehensive response to HIV/AIDS, prepared by UNAIDS (Gutierrez JP, written communication). Kenya is in the middle of the range. Drop out rate on ART varies from 5% (optimistic scenario) to 20% [10][22].
ART, people without TB	Person year of treatment	640	Uniform (487, 743)	Sources and assumptions as stated above for ART for TB patients.

Finally, we added calculation of the number of disability-adjusted life years (DALYs) gained to the measures of effectiveness that the model was initially designed to consider (i.e. TB cases prevented and TB deaths averted). The calculation of DALYs gained was important because the health gains from ART and HIV prevention strategies are broader than prevented TB cases and deaths; thus, fair comparisons among the seven strategies that we considered required a more generic measure of effectiveness. We assumed that averting a TB death would lead to a gain of 4 DALYs in an HIV-positive person and 24 DALYs in an HIV-negative person [26-30]. We assumed that one DALY is gained for each year that a person takes ART plus one DALY for each year that the person survives after defaulting from ART. We assumed that for each HIV infection averted, 22 DALYs are gained[7,26,27]. DALYs gained in future years were discounted at 3% per annum.

We considered each intervention separately, using Monte Carlo simulations to obtain probability distributions and 95% confidence intervals for model outputs (see [10] for further details). Total costs and effects are incremental costs and effects, i.e. the net increase or decrease in costs or change in effects as compared to the baseline scenario. Cost-effectiveness was calculated as the net change in costs divided by the net change in effects, both as compared to the baseline scenario; we report the cost per TB case averted, per death averted and per DALY gained.

The population level impact on HIV of interventions aimed at reducing HIV incidence is difficult to quantify, because the effect of HIV prevention interventions on HIV incidence has seldom been quantified. The few randomized controlled trials that have been done lead to contradictory conclusions[31,32]. We therefore estimated the cost per HIV infection averted at which the cost-effectiveness of HIV prevention matched that of the other strategies being considered.

## Results

A summary of the major cost, effectiveness and cost-effectiveness results is provided in Table 3.

**Table 3:** Summary of major cost, effectiveness and cost-effectiveness results for the strategies assessed, 2004–2023*

**Total annual costs (US$ millions)**					
Intervention	TB Treatment	AIDS Treatment, excluding ART	ART or TLTI	Total Annual Costs	Total Incremental Cost vs. Baseline Scenario

Baseline scenario†	18.5	63.7	0.0	82.2	N/A
Improve TB Detection Rate	27.4	66.7	0.0	94.1	11.9
Improve TB Cure Rate	25.1	64.7	0.0	89.8	7.6
Improve TB Cure Rate and Case Detection Rate	26.9	67.3	0.0	94.2	12.0
TLTI, 6 months	18.3	63.7	0.7	82.8	0.6
TLTI, Lifetime	18.0	63.6	8.0	89.7	7.5
ART, 50% dropout rate	18.2	62.1	153	233	151
ART, 20% dropout rate	17.5	58.8	348	424	342
ART, 5% dropout rate	16.2	52.1	656	725	642
ART to TB patients	18.4	62.5	71.7	152.6	70.5

**Total annual effects‡**					

Intervention	DALYs Gained vs. Baseline Scenario		Deaths Averted vs. Baseline Scenario		TB Cases Averted vs. Baseline Scenario

Baseline scenario†	N/A		N/A		N/A
Improve TB Detection Rate	551,184		26,015		59,436
Improve TB Cure Rate	220,939		10,087		43,378
Improve TB Cure Rate and Case Detection Rate	676,748		31,769		97,795
TLTI, 6 months	7,366		549		2,119
TLTI, Lifetime	20,178		2,075		5,480
ART, 50% dropout rate	585,232		7,390		4,025
ART, 20% dropout rate	834,071		23,794		12,183
ART, 5% dropout rate	1,205,912		56,872		27,446
ART to TB patients	152,604		5,089		1,126

**Cost-effectiveness (US$)§**					

Intervention	Cost per DALY Gained		Cost per Death Averted		Cost per TB Case Averted

Baseline scenario†	N/A		N/A		N/A
Improve TB Detection Rate	21.6		458		201
Improve TB Cure Rate	34.3		752		175
Improve TB Cure Rate and Case Detection Rate	17.8		379		123
TLTI, 6 months	84.7		1,136		294
TLTI, Lifetime	373		3,630		1,374
ART, 50% dropout rate	258		20,461		37,569
ART, 20% dropout rate	410		14,370		28,064
ART, 5% dropout rate	533		11,294		23,403
ART to TB patients	462		13,846		62,578

*Total incremental costs and total incremental effects are average figures across the 20 year period that was considered. Trends over time are shown in the figures.

† The baseline scenario is defined as TB treatment only, with a case detection rate of 50% and a cure rate of 70%, and no TLTI or ART.

‡ In the baseline scenario there are an estimated 774,000 deaths per year and an estimated 225,000 TB cases per year

§Calculated as incremental costs divided by total incremental effects. Numbers are sometimes different to the total costs divided by total effects shown in the table due to rounding.

**Figure 3:**
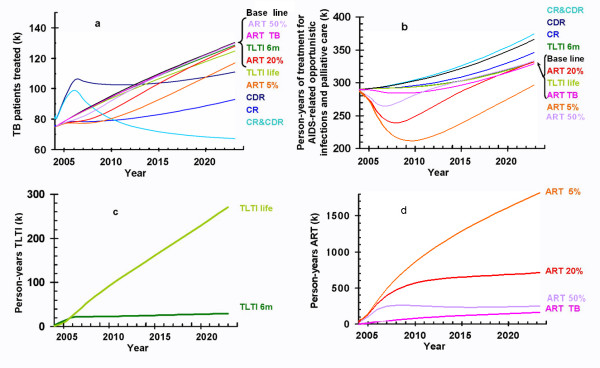
Numbers receiving treatment for different strategies: (a) number of TB patients treated; (b) number of person years of AIDS-related opportunistic infection treatment and palliative care (not including ART); (c) number of person years of TLTI; (d) number of person years of ART. CDR = Case detection rate. CR = Cure rate. 6 m = 6 months. ART 50%, ART 20% and ART 5% mean ART with a 50%, 20% and 5% drop out rate, respectively. ART TB is ART for TB patients only, at a dropout rate of 20%. (k) means thousands.

In the baseline scenario (i.e. 50% TB case detection rate and 70% cure rate), the number of TB patients treated steadily increases (Figure 3a). The trend is similar when TLTI is provided or if ART is available with a drop out rate of 50% or 20% per year. Providing ART only has a noticeable long-term impact on the number of TB patients treated if the drop out rate is as low as 5% per year. Improving TB cure rates, and simultaneously improving TB case detection and cure rates, both cause a substantial reduction in the number of TB patients treated. Improving TB case detection has less impact, but by 2012 it reduces the number of TB patients treated compared to the baseline scenario.

The number of person-years of treatment for AIDS-related opportunistic infections and palliative care for people with AIDS (i.e. AIDS-related treatment and care other than ART) steadily increases in the baseline scenario (Figure 3b). Only the provision of ART leads to a substantial reduction (Figure 3b), provided the drop out rate per year is relatively low.

If TLTI is provided for six months, the number of people receiving TLTI stabilizes at about 25,000 per year; if it is provided for life the number reaches 150,000 after 10 years and almost 300,000 after 20 years (Figure 3c). If ART is given to 50% of those in need and the drop out rate is 20%, the number of people receiving ART will reach 700,000 by 2024 (Figure 3d). However, if the drop out rate is 5% per year the number of people receiving ART will reach almost 2 million by 2024.

**Figure 4:**
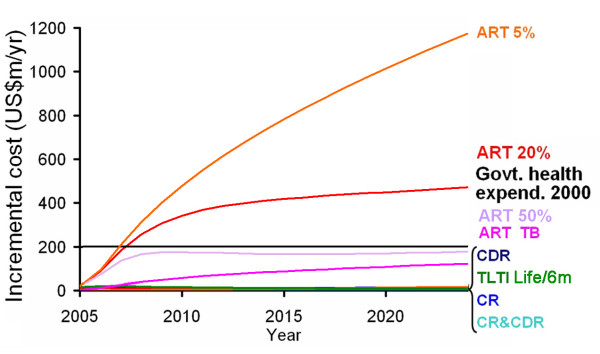
Incremental costs compared to baseline scenario for each strategy. Black line represents estimated total government health expenditure in Kenya in the year 2000. See Figure 3 text for label definitions.

The incremental costs (i.e. the net increase in cost compared to the baseline scenario) of the different strategies are shown in Figure 4. The additional annual cost of improving TB cure rates rises to US$7.5 million ten years after improvements are first implemented. Beyond 2013 costs increase more slowly, reaching a peak of US$8.6 million after twenty years. The additional annual cost of improving TB case detection rates increases sharply to US$12 million after ten years and then remains fairly steady (between US$12 million and US$14 million) for the rest of the twenty years. Improving TB case detection and cure rates simultaneously is initially more costly than implementing either strategy independently, with a peak incremental cost of US$12 million in 2013. From then onwards, additional annual costs decrease year-on-year as transmission and hence the number of TB cases are reduced, falling to just under US$10 million per year after 20 years. Providing ART is considerably more expensive than the other strategies. With a drop out rate of 20% per year, the incremental cost reaches $200 million per year in 2007 (equal to total government health expenditure in 2000); with a drop out rate of 5% per year the incremental cost reaches US$200 million in 2006 and US$1 billion in 2023. With a drop-out rate of 50% per year, the total cost reaches the level of government health expenditures in year 2000 by around 2009. The incremental cost of providing TLTI is much lower even if it is offered for life.

**Figure 5:**
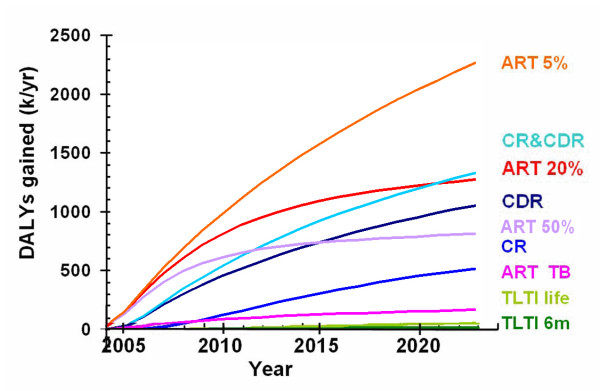
Disability adjusted life years (DALYs) gained by each strategy. See Figure 3 text for label definitions.

Figure 5 shows the incremental effectiveness of each strategy (i.e. the net increase or decrease in DALYs gained compared to the baseline scenario). Providing ART with a drop out rate of 5% per year is the most effective strategy and could gain an extra 2.3 million DALYs each year by 2024. Providing ART with a drop out rate of 20% per year gains about 1.3 million extra DALYs per year by 2024. In the long run, simultaneous improvement of TB case detection and cure rates gains slightly more DALYs per year than providing ART with a 20% per year drop out rate, although ART averts more when averaged over the twenty years. TLTI gains a comparatively small number of extra DALYs.

**Figure 6:**
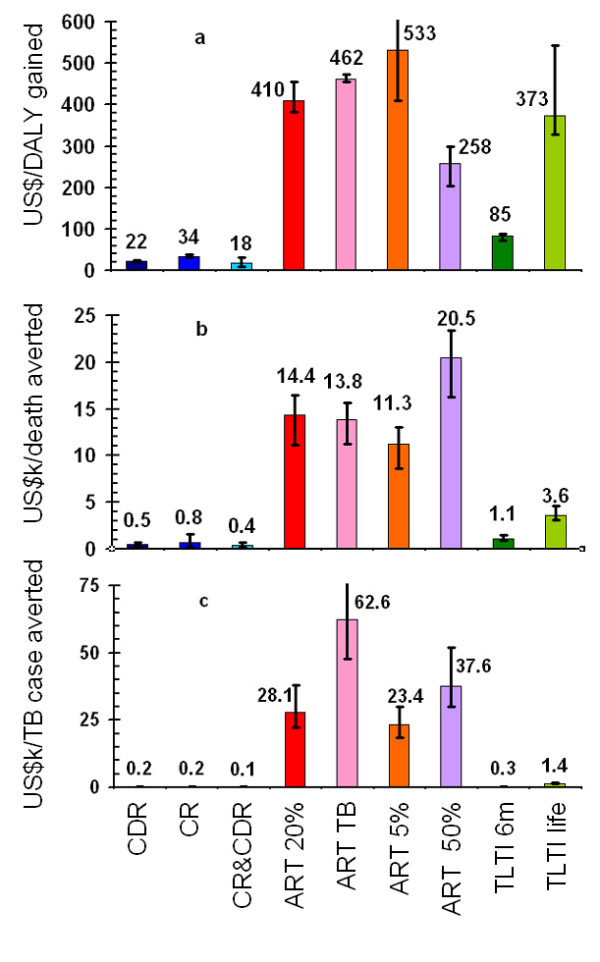
Cost-effectiveness of each strategy, with 95% confidence intervals. See Figure 3 text for label definitions.

Figure 6 shows the cost-effectiveness of the different interventions. Improving TB case detection costs US$22 per DALY gained, and improving cure rates costs US$34 per DALY gained. Doing both simultaneously costs US$18 per DALY gained. ART costs around US$260 to US$530 per DALY gained, depending on the drop-out rate and the type of patients considered (people enrolled as TB patients only, or all people enrolled on ART). To gain a DALY by providing TLTI for 6 months, at US$85, is more than four times as costly as improving TB cure rates and detection rates simultaneously and TLTI for life has a similar cost to ART, at US$373 per DALY gained. The cost per death averted is less than US$800 for the TB interventions, while providing ART costs around US$11,000 to US$20,000 to avert one death. Providing TLTI for 6 months does rather better at about US$1,100 to avert one death. The cost per TB case averted is also small for improvements in TB case detection and cure rates (about US$ 200 per case averted or less), while providing ART costs about US$30,000 to US$60,000 per TB case averted.

**Figure 7:**
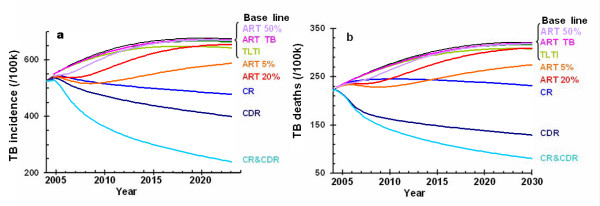
Effect of each strategy on TB incidence and TB deaths: (a) TB incidence per 100,000 population; (b) TB deaths per 100,000 population. See Figure 3 text for label definitions.

Figure 7 shows the impact of the various interventions on TB incidence and death rates. Providing ART or TLTI has little impact on TB incidence or deaths. Improving TB cure rates leads to a slow but steady decline in TB incidence and deaths, improving case detection rates does rather better and doing both simultaneously leads to TB cases and deaths declining by almost 50% between 2004 and 2023. However, compared with estimated TB incidence and death rates of, respectively, 104 and 49 per 100,000 population in 1990 (the baseline year to which the Millennium Development Goals refer), the decline in both cases and deaths would be much smaller.

Taking the costs of other interventions averaged over ten years, averting one HIV infection for less than US$4,000 to US$5,000 would be as cost effective as ART and averting one HIV infection for less than US$250 would be as cost effective as improving TB case detection and cure simultaneously to WHO target levels.

Full uncertainty analysis results are available from authors upon request but the major results over time periods of 10 years and for HIV prevalence stabilising at 7% are produced in Tables 4 and 5. In brief, these show that the relative cost-effectiveness of the different strategies is similar when a time period of ten years is considered, and when HIV prevalence stabilizes at 7%. The pattern is also similar when the rate of progress towards targets is 25% and 50% of the level considered in this article (data not shown). There are two main differences worth noting. The first is that cost-effectiveness improves for strategies that have an important impact on TB transmission when a longer time period is considered (for example, the cost per DALY gained by simultaneously improving TB case detection and cure rates is US$18 when a time period of 20 years is considered, and US$37 when a time period of 10 years is considered). The second is that when HIV prevalence is assumed to stabilize at 7%, the numbers receiving TLTI and ART are reduced by about 50%, as would be expected. As a consequence, improving the TB case detection rate averts as many DALYs as ART with a 5% annual dropout rate, and simultaneous improvement in TB case detection and cure rates gains the largest number of DALYs.

**Table 4:** Summary of major cost, effectiveness and cost-effectiveness results for the strategies assessed, 2004–2013*

**Total annual costs (US$ millions)**					
Intervention	TB Treatment	AIDS Treatment, excluding ART	ART or TLTI	Total Annual Costs	Total Incremental Cost vs. Baseline Scenario

Baseline scenario†	16.3	61.8	0.0	78.2	N/A
Improve TB Detection Rate	26.3	63.1	0.0	89.4	11.2
Improve TB Cure Rate	23.4	62.1	0.0	85.5	7.3
Improve TB Cure Rate and Case Detection Rate	28.3	63.3	0.0	91.7	13.5
TLTI, 6 months	16.2	61.8	0.646	78.7	0.6
TLTI, Lifetime	16.1	61.8	4.13	82.1	3.9
ART, 50% dropout rate	15.9	59.0	143	217	139
ART, 20% dropout rate	15.2	54.5	277	346	268
ART, 5% dropout rate	14.4	49.6	409	473	395
ART to TB patients	16.2	60.6	47.45	124	46.1

**Total annual effects‡**					

Intervention	DALYs Gained vs. Baseline Scenario		Deaths Averted vs. Baseline Scenario		TB Cases Averted vs. Baseline Scenario

Baseline scenario†	N/A		N/A		N/A
Improve TB Detection Rate	307,158		19,034		34,193
Improve TB Cure Rate	85,223		5,005		24,447
Improve TB Cure Rate and Case Detection Rate	362,939		22,356		58,268
TLTI, 6 months	2,924		346		1,244
TLTI, Lifetime	5,952		836		2,535
ART, 50% dropout rate	444,020		14,313		5,698
ART, 20% dropout rate	578,943		36,105		13,930
ART, 5% dropout rate	706,577		59,075		22,405
ART to TB patients	86,835		5,714		1,045

**Cost-effectiveness (US$)§**					

Intervention	Cost per DALY Gained		Cost per Death Averted		Cost per TB Case Averted

Baseline scenario†	N/A		N/A		N/A
Improve TB Detection Rate	37		591		329
Improve TB Cure Rate	86		1,470		300
Improve TB Cure Rate and Case Detection Rate	37		604		232
TLTI, 6 months	190		1,610		447
TLTI, Lifetime	655		4,660		1,540
ART, 50% dropout rate	314		9,730		24,400
ART, 20% dropout rate	463		7,420		19,200
ART, 5% dropout rate	559		6,690		17,600
ART to TB patients	531		8,070		44,100

*Total incremental costs and total incremental effects are average figures across the 10 year period that was considered. Trends over time are shown in the figures.

† The baseline situation is defined as TB treatment only, with a case detection rate of 50% and a cure rate of 70%, and no TLTI or ART.

‡ In the baseline scenario there are an estimated 725,000 deaths per year and an estimated 198,000 TB cases per year

**§**Calculated as incremental costs divided by total incremental effects. Numbers are sometimes different to the total costs divided by total effects shown in the table due to rounding.

**Table 5:** Summary of major cost, effectiveness and cost-effectiveness results for the strategies assessed if the predicted HIV prevalence is halved, 2004–2023*

**Total annual costs (US$ millions)**					
Intervention	TB Treatment	AIDS Treatment, excluding ART	ART or TLTI	Total Annual Costs	Total Incremental Cost vs. Baseline Scenario

Baseline scenario†	17.6	32.3	0.0	49.8	N/A
Improve TB Detection Rate	24.3	34.2	0.0	58.4	8.6
Improve TB Cure Rate	23.8	32.9	0.0	56.7	6.8
Improve TB Cure Rate and Case Detection Rate	23.5	34.6	0.0	58.1	8.3
TLTI, 6 months	17.5	32.3	0.401	50.2	0.3
TLTI, Lifetime	17.3	32.3	4.43	54.0	4.2
ART, 50% dropout rate	17.3	31.4	80.3	129.0	79.2
ART, 20% dropout rate	16.7	29.6	180	226.8	176.9
ART, 5% dropout rate	15.7	26.2	337	378.7	328.9
ART to TB patients	17.5	31.5	45.84	94.8	45.0

**Total annual effects‡**					

Intervention	DALYs Gained vs. Baseline Scenario		Deaths Averted vs. Baseline Scenario		TB Cases Averted vs. Baseline Scenario

Baseline scenario†	N/A		N/A		N/A
Improve TB Detection Rate	662,609		28,344		66,677
Improve TB Cure Rate	268,347		11,258		45,780
Improve TB Cure Rate and Case Detection Rate	816,465		34,826		106,872
TLTI, 6 months	6,036		367		1,473
TLTI, Lifetime	16,193		1,120		3,801
ART, 50% dropout rate	311,693		4,566		3,496
ART, 20% dropout rate	453,396		14,214		10,485
ART, 5% dropout rate	662,802		32,773		23,189
ART to TB patients	100,602		3,513		1,360

**Cost-effectiveness (US$)§**					

Intervention	Cost per DALY Gained		Cost per Death Averted		Cost per TB Case Averted

Baseline scenario†	N/A		N/A		N/A
Improve TB Detection Rate	13		303		129
Improve TB Cure Rate	25		606		149
Improve TB Cure Rate and Case Detection Rate	10		237		77
TLTI, 6 months	52		862		215
TLTI, Lifetime	257		3,710		1,090
ART, 50% dropout rate	254		17,300		22,700
ART, 20% dropout rate	390		12,400		16,900
ART, 5% dropout rate	496		10,000		14,200
ART to TB patients	447		12,800		33,100

*Total incremental costs and total incremental effects are average figures across the 20 year period that was considered. Trends over time are shown in the figures.

† The baseline situation is defined as TB treatment only, with a case detection rate of 50% and a cure rate of 70%, and no TLTI or ART.

‡ In the baseline scenario there are an estimated 648,000 deaths per year and an estimated 224,000 TB cases per year

§Calculated as incremental costs divided by total incremental effects. Numbers are sometimes different to the total costs divided by total effects shown in the table due to rounding.

## Discussion

This study suggests that the most cost-effective and affordable strategies for controlling TB in Kenya are to further increase TB case detection and cure rates, preferably in combination. Providing ART at the levels proposed in the WHO's "3-by-5" initiative has limited impact on TB incidence and death rates, but because it prevents deaths from both TB and non-TB related causes it could gain more DALYs than would be achieved by reaching global TB control targets if the drop out rate is low. High compliance with ART is clearly desirable, and as the cumulative number of people on ART builds up will require investment that is substantial in the context of existing levels of government health expenditure. In contrast, TLTI has relatively low total costs and effects. Similar findings are likely to apply to other countries with HIV and TB epidemics that are at a similar stage to those in Kenya i.e. countries where rapid increases in HIV and TB incidence have already occurred, where income levels and hence unit costs are comparable, and where HIV prevalence is now stable or starting to fall.

The effectiveness of improvements in TB case detection and cure rates in terms of DALYs gained may appear surprisingly large, especially compared to ART. One explanation is that improvements in TB case detection and cure rates result in DALYs being gained among both HIV-positive and HIV-negative individuals. With HIV prevalence among TB patients about 30%, an average of 18 DALYs are gained for every TB death that is averted. Under our assumptions this is equivalent to the gain from 18 years of ART. The second explanation is that improvements in TB control have a major effect on TB transmission.

ART has a relatively small impact on TB outcomes because we have assumed, in line with the WHO's "3-by-5" Initiative, that the people in need of ART are those HIV-positive people who, without access to treatment, would be expected to die within two years. To have a major impact on TB incidence and deaths, ART would need to be given earlier in the course of HIV-infection[15]. The effectiveness of TLTI is limited by coverage of HIV testing and counselling services. We have assumed that people will only be given TLTI if their HIV status is known and active TB has been excluded. Recent pilot projects suggest that there is a relatively low level of uptake and completion[21]. However, providing TLTI for just 6 months appears to be relatively cost-effective. The limited additional benefits and large costs of providing TLTI for life suggest that this should not be promoted as a public health intervention.

Previous studies suggest that condom distribution programmes cost US$11 to US$2,000 per HIV infection prevented[7]. Alternatively, if we assume that the risk of HIV infection per sex act varies from about 1 per 100 sexual encounters among those at highest risk to 1 per 1,000 sexual encounters among those at medium risk[33] and that the cost of providing a condom is US$0.25, then the cost of averting one HIV infection by making condoms available is between US$25 and US$250 per infection averted. Although these estimates cover a rather broad range, comparison with our threshold analyses suggest that HIV prevention programmes can compete favourably with the other strategies that we considered.

This analysis has some limitations. First, the benefits of ART could have been under or over-estimated. Under-estimation is possible because the epidemiological model does not allow for the impact that ART may have on HIV transmission or TB cure rates. ART may reduce transmission when it is started at relatively high CD4+ cell counts, as in developed countries[34]. Current guidelines for resource-poor settings such as Kenya recommend starting ART later at lower CD4+ cell counts[14], at which there is no evidence that ART will reduce overall levels of transmission. Nevertheless, ART may offer opportunities to strengthen prevention efforts quite apart from its impact on lowering the viral load in people who access treatment[35], and it is also possible that it will increase TB cure rates by improving the general health of TB patients. Overestimation is possible because we assumed that one DALY would be gained for each year of ART. This is equivalent to assuming that there is no morbidity associated with ART. In practice, some people on ART will experience side-effects.

Second, the costs of providing ART are difficult to assess. In particular, the non-drug costs that we used are based on the strategy developed for achievement of the "3-by-5" goal [5,36], which places emphasis on community-based care and limited use of CD4 count and viral load tests. Drug costs are already low and may not fall much further, but other costs could have been underestimated because there is little evidence about the costs of providing ART in practice and on a massive scale in poor countries. More documentation of the costs of existing ART programmes, especially those that are now being scaled up nationwide, is needed. If ARV drug costs are assumed to be zero (as opposed to around US$140 per year), our analyses suggest that the cost per DALY gained by ART would be about US$320 to US$420.

Third, few data have been published on the life expectancy of patients who default from ART. We have assumed that following default a person is at the same stage in the natural history of HIV as someone entering late-stage HIV (WHO stage 3). This means that those given ART effectively pass through stage 3 twice: first before being given ART; second following default from ART. During stage 3, they are more susceptible to TB, and therefore individuals that are HIV-positive and given ART have an increased risk of TB for longer than HIV-positive individuals not given ART. There are insufficient data available to confirm whether this is an appropriate assumption, but this may explain why the reduction in the number of TB cases under the ART strategy described in this study is even lower than that predicted elsewhere[15].

Fourth, we only assessed the impact of implementing one strategy at a time. It will be of importance to explore the combined effect of TB control and the provision of ART, which together are likely to have a bigger impact on both TB and HIV.

Fifth, there is as yet limited evidence about the costs of improving TB case detection and cure rates in practice, once coverage of the DOTS strategy has reached 100%. We combined data about the existing cost per patient detected and treated[11] with available data on the factor by which the average cost per patient detected and treated will change as case detection and cure rates are improved. While we have used estimates of the factor by which average costs change, our results are consistent with the proposed total increase in Kenya's TB control budget included in recent data submitted to WHO and in the country's recent funding applications to the GFATM. Our results suggest an increase in annual costs of around US$12 million, compared to the proposed budgetary increase of US$11.5 million. Evidence about the relationship between increased spending and improvements in case detection and cure rates should be collected in the next few years in Kenya and other countries, to assist planning and budgeting for improved TB control and so that the relationship between costs and improved case detection and cure rates can be better understood.

Our findings support current efforts in Kenya to increase TB case detection rates and improve cure rates. Recent analyses suggest that this is feasible through measures such as expanding DOTS implementation beyond the 35% of health facilities in which it is currently available, expanding DOTS implementation into the private sector, extending existing training to nurses who are the first contact with the health system for many TB suspects and who play a major role in delivery of TB treatment, and through implementation of advocacy, communication and social mobilization activities (C. Hanson, personal communication; T. Pennas, written communication).

## Conclusion

Overall, our study suggests that the priority for national TB control programmes in high HIV prevalence settings should be to concentrate first on improving TB cure and case detection rates. This should be highly cost-effective, affordable in the context of existing national health budgets, and could make an important contribution to achievement of the Millennium Development Goal targets related to TB. Realising the full potential of ART will require substantial new funding and strengthening of health system capacity so that increased funding can be used effectively.

## Competing interests

The author(s) declare that they have no competing interests.

## Authors' contributions

All authors contributed to the conception and design of the study. CC took primary responsibility for the analysis, with guidance on mathematical modelling provided by BG and CD, and guidance on economic analysis provided by KF. CC wrote the first draft of the paper; BG and KF finalized the paper with input from CD. All authors approved the final version.

## Pre-publication history

The pre-publication history for this paper can be accessed here:


